# Identifying leverage points to sustain community-engaged research infrastructure in academic systems: a participatory systems science approach

**DOI:** 10.3389/fpubh.2026.1970793

**Published:** 2026-10-21

**Authors:** Lan N. Đoàn, Rachel L. Thompson, Sze Wan Chan, Perla Chebli, Stella S. Yi

**Affiliations:** 1 Department of Population Health, NYU Langone Health, New York, NY, United States; 2 Center for Systems and Community Design, City University of New York Graduate School of Public Health and Health Policy, New York, NY, United States

**Keywords:** community health, community-based participatory research, community-engaged research, group model building, systems oriented approach, systems thinking

## Abstract

**Background:**

This study aimed to characterize multi-level factors influencing sustainability of community-engaged research (CEnR) infrastructure in academic systems and identify intervention leverage points.

**Methods:**

We used a participatory systems science approach. Interviews with 28 principal investigators and 20 study staff informed an initial causal loop diagram, which was refined in group model building workshops to identify strategies to improve CEnR practices. An impact and feasibility survey and plenary session were used to prioritize strategies.

**Results:**

Interacting feedback loops shaped CEnR sustainability across three domains: funding, institutional barriers, and authentic partnerships, with academic culture influencing all three. Participants identified 27 strategies to improve CEnR practices. Prioritized strategies included relationship building, hiring community members, expanding funding for community partners, diversifying grant review panels, broadening evaluation beyond quantitative metrics, and implementing flexible payment processes.

**Discussion:**

Sustaining CEnR requires changes in academic culture, funding structures, and institutional practices. Systems-informed strategies that reduce administrative burden, value community-responsive outcomes and relationships, and invest in authentic partnerships may strengthen CEnR sustainability. Academic institutions and funders can support CEnR by aligning promotion, evaluation, and financial policies with community-engaged work.

## 1 Introduction

Community-engaged research (CEnR) includes partnership models from outreach to co-leadership, including community-based participatory research (CBPR) and participatory action research (1). These approaches have advanced community engagement in clinical research and translational science (2). In 2022, the National Academy of Medicine emphasized that meaningful community engagement requires equal partnerships and bidirectional communication, with core indicators that include increased knowledge, strengthened alliances, improved health programs and policy, and thriving communities (3). Yet challenges – including historical mistrust in research, fiscal and administrative barriers in academia, power imbalances, and tensions around data ownership and governance – continue to hinder effective CenR (1, 4). While existing frameworks have identified key determinants of CEnR implementation, evaluation, and sustainability, they have not mapped specific structural processes or the interrelated nature of these processes that shape CEnR over time in academic settings (1, 5).

Prior work has emphasized institutional champions, strict adherence to collaborative principles during funding transitions, and tools like Memorandum of Understandings (MOUs) for preventing unilateral decision-making (6, 7). Recent research emphasizes equitable power-sharing, documented community benefit, co-creation, and small grant programs that build trust and leverage resources beyond initial grant cycles (8, 9). Similar institutional and funding challenges have also been identified in the co-production literature in sustainability and environmental sciences, where parallel reforms to academic systems and funding mechanisms have been highlighted to support justice-oriented and community-engaged research (10, 11).

Despite these advances, persistent challenges remain, including resource constraints, role ambiguity, and the perceived extractive nature of academia (9). Recurring barriers over two decades include rigid institutional fiscal policies and the time required for relationship building, which often conflicts with academic productivity expectations (8). Longitudinal prospective evaluation also remains limited, with few standardized quantitative tools to assess the long-term effects of collaborative involvement on research relevance and impact (12, 13).

These multi-layered challenges reflect that CEnR operates within a broader academic research ecosystem shaped by dynamic interactions between funding agencies, academic institutions, communities and community organizations, and broader policy and political contexts. This ecosystem influences which research questions are prioritized, how partnerships are sustained, and which forms of knowledge and impact are recognized. A systems perspective is therefore useful for understanding the interdependencies that shape CEnR and for identifying leverage points to improve its sustainability. Systems science approaches are well suited for examining such complexity, and participatory methods such as group model building (GMB) can help diverse interest-holders develop a shared understanding of problems and identify actionable strategies for systems change (14).

We focus on leverage points for impactful CEnR in academic settings because academic institutions often govern the policies and practices that condition community engagement. Examining the multi-level drivers of long-term CEnR in these settings is therefore critical for identifying organizational strategies that can shift policies and practices within existing structures and power dynamics. Drawing on the expertise of research teams working with communities, we used a participatory systems science approach to characterize factors contributing to sustained CEnR infrastructure in academic systems, identify leverage points, and develop recommendations to support CEnR.

## 2 Methods

### 2.1 Participatory systems science framework

This project drew on principles of system dynamics modeling, a systems science approach used to understand complex systems and identify leverage points for systems change (15). We incorporated elements of GMB, a participatory approach rooted in community-based participatory research (14). Through GMB workshops, interest-holders collaborate with modelers and subject matter experts to co-construct a shared understanding of the forces driving a complex problem (14).

Causal loop diagrams (CLDs) are visual tools used in system dynamics to represent how variables within a system influence one another and are connected through feedback loops (15). CLDs consist of three primary components: variables, arrow linkages, and feedback loops. Arrow linkages show the hypothesized cause-and-effect relationships between variables, where positive (+) links indicate change in the same direction, and negative (−) links indicate change in opposite directions. When arrows form a closed loop, they create feedback; reinforcing loops (R) drive growth or decline, while balancing loops (B) bring stability to the system (see Supplementary File 1).

While system dynamics methods can generate powerful insights into complex systems, they can also be resource- and time-intensive, and result in models that are theoretical and not actionable or feasible in real-world settings (16). Therefore, we used an adapted GMB approach where a preliminary CLD derived from prior interview data was refined through workshops, rather than constructing a CLD from scratch during workshops.

### 2.2 Participant recruitment

Recruitment processes for the prior interviews that informed this study are described elsewhere (17). Briefly, we used purposive and snowball sampling to identify principal investigators (PIs) and study staff with experience engaging communities experiencing health disparities from research institutions in the United States. We focused on research involving the 14 National Institutes of Health (NIH)-designated populations that experience health disparities, including communities of color, women, people with disabilities, populations with low socioeconomic status, underserved rural populations, and sexual minority groups, as well as the intersections among these groups (18).

Participants were eligible to participate in GMB sessions if they had previously completed a study interview (17), were aged 18 years and older, and provided informed consent. At the end of the interviews, participants were asked if they were interested in participating in a GMB workshop. Those interested were re-contacted by email to confirm willingness to participate, and those who agreed received a consent form to review and a Zoom meeting link. Verbal consent was obtained at the start of each GMB workshop. The same process was used for PIs and study staff. The NYU Langone Health Institutional Review Board approved the study protocol.

### 2.3 Development of the initial CLD

An initial CLD “seed model” was developed using findings from interviews with 48 research team members (28 PIs and 20 study staff) conducted between April 2024 and March 2025 (17). Interview questions explored experiences with and perspectives on CEnR, including perceived challenges and opportunities. Interviews were audio-recorded with consent, and interview notes were analyzed using thematic analysis to identify key themes related to conducting CEnR, including sustainability. These themes informed the GMB design, study problem statement, and development of the initial CLD. Detailed interview methods and findings are reported elsewhere (17).

### 2.4 GMB workshops

We conducted separate GMB workshops in September 2024 for PIs and study staff to minimize the potential influence of power dynamics. Participants received $150 for one 2-h workshop. Before each workshop, participants received the initial CLD developed from interview analysis (Figure 1A) and a guide on interpreting CLDs (Supplementary File 1).

**Figure 1:**
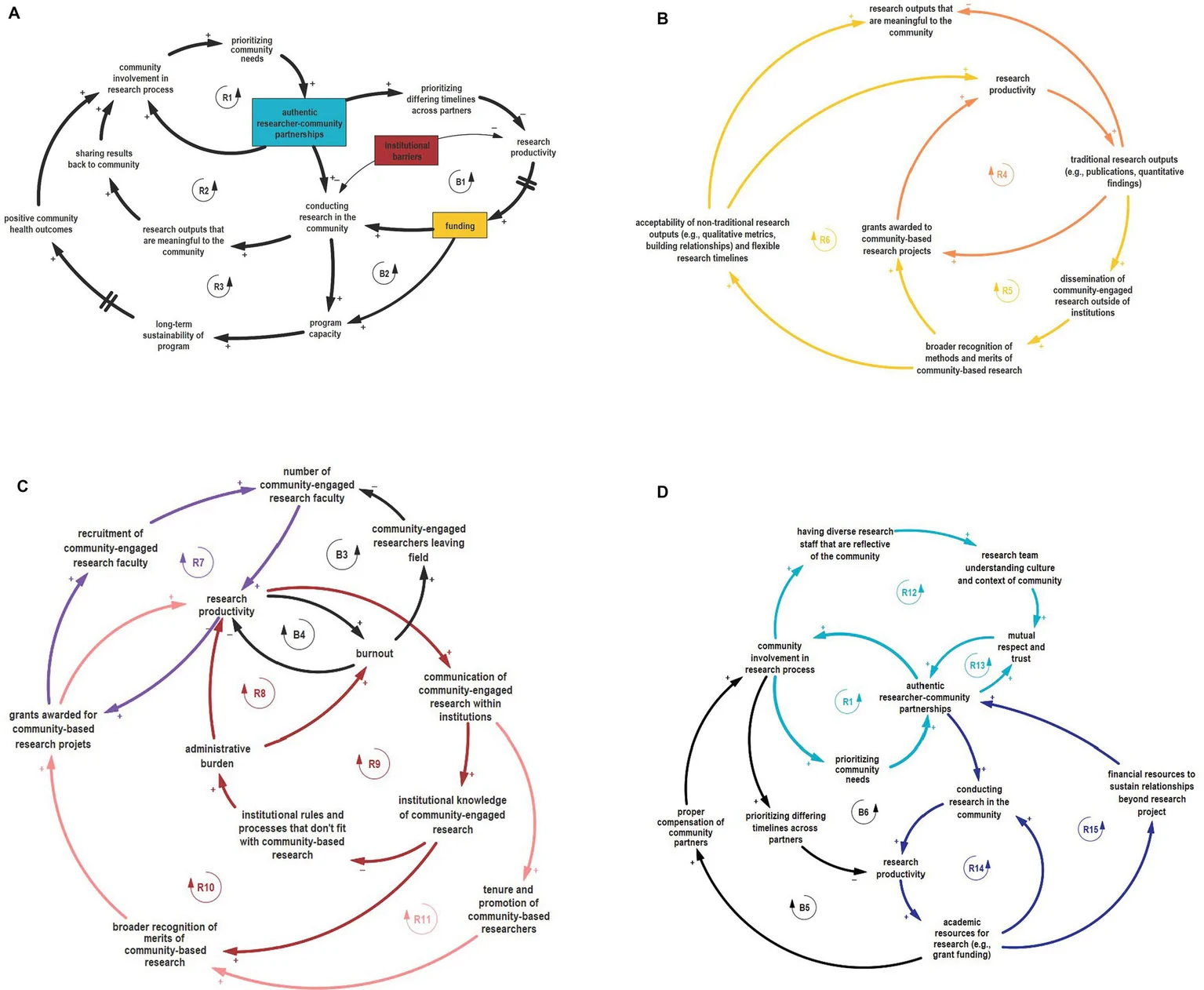
Causal loop diagrams of academic systems influencing community-engaged research. **(A)** Preliminary CLD developed from interview analysis shared with participants prior to GMB workshops. The three boxed variables (*funding*, *institutional barriers*, and *authentic partnerships*) represent focal domains selected for subsequent GMB activities, resulting in the loops presented in **(B–D)**. The lighter-intensity arrows from the *institutional barriers* variable outside the connected loops indicate that institutional barriers were originally conceptualized as exogenous influences on the on the system. **(B)**: Funding feedback loops. **(C)** Institutional barriers feedback loops. **(D)** Authentic community-academic partnerships feedback loops. Reinforcing loops (R) indicate processes that amplify change over time and balancing loops **(B)** indicate processes that counter change and promote stability. Positive (+) linkages indicate variables that change in the same direction, and negative (−) linkages indicate variables that change in opposite directions. In **(B–D)**, loops of the same color represent a thematic grouping, described together in the main text.

Both workshops followed the same agenda. Participants reviewed the problem statement, session goals, initial CLD “seed model,” and were introduced to systems thinking concepts (e.g., CLD components and feedback loops). Participants were then asked to reflect on the CLD, ask questions, and identify missing or incorrect variables. Lastly, participants responded to discussion prompts within three *a priori* domains—funding, institutional barriers, and authentic partnerships, with academic culture considered as a cross-cutting domain—to generate recommendations and identify leverage points for strengthening CEnR infrastructure. For the funding domain, participants were asked:

*“What are your recommendations for how funding mechanisms can be modified to fit the nature of community-engaged research,”* and

*“How can academic culture be modified to support funding mechanisms that fit the nature of community-engaged research?”*

For the institutional barriers domain, participants were asked:

*“What are your recommendations for how research institutions can better support the conduct of community-engaged research?,”* and

*“What changes should be made to academic culture to remove institutional barriers to the conduct of community-engaged research?”*

To stimulate discussion within this domain, participants were provided with examples of institutional barriers that had emerged from the interview analysis, including institutional review board (IRB) requirements, incentive restrictions, and human resources (HR) hiring policies that may hinder effective CEnR. Finally, for the authentic partnerships domain, participants were asked:

*“How do we maintain long-term relationships with underrepresented communities?,”*

*“How do we sustain researcher-community partnerships beyond the duration of a specific research project?,”* and

*“How can academic culture be modified to support relationships between researchers and underrepresented communities?”*

Ideas were captured as sticky notes on a Microsoft Whiteboard and the research team took detailed notes and collected other cognitive artifacts to capture participant contributions, recommendations, and decision-making processes.

#### 2.5 Feasibility and impact survey

Recommendations and strategies identified from GMB workshops were compiled into a REDCap (Research Electronic Data Capture) survey. The survey was sent to GMB participants and interviewed participants who had expressed interest in a GMB workshop (*n* = 36 potential participants) in January 2025. Strategies were organized by domain (funding, institutional barriers, authentic partnerships) and level of influence (researcher, institution, funder, academic culture). Participants were asked to rate the perceived feasibility and impact of each strategy and rank its priority within each domain.

#### 2.6 Plenary session

A final plenary GMB session was conducted in January 2025 to review the revised CLDs, gather additional feedback, and prioritize strategies identified during the earlier workshops. Plenary participants included GMB participants and all interviewees who were interested in GMB participation. During the session, the research team presented the updated CLDs and facilitated a structured ranking activity to identify the highest-priority recommendations across domains. Participants received a $50 gift card.

#### 2.7 Synthesis of findings

Findings from the interviews, GMB workshops, plenary session, and impact and feasibility survey were integrated to produce the final study outputs. CLDs were developed and refined using Vensim software^1^ and finalized after review of session notes and incorporation of missing variables and links. Recommendations were compiled, de-duplicated, and organized by domain. For the impact and feasibility survey, participants rated each strategy on perceived feasibility and perceived impact using a five-point scale, with higher scores indicating greater perceived feasibility or impact. Mean perceived feasibility and impact scores were calculated for each strategy, and feasibility was plotted against impact to identify potential leverage points. Quantitative analyses and visualizations were conducted in R software.^2^

## 3 Results

A detailed breakdown of study activities and participant involvement is provided in Table 1. Interview data from 28 PIs and 20 CEnR study staff (*n* = 48) informed development of an initial CLD and subsequent GMB workshops (17). Two GMB workshops were conducted separately with PIs (*n* = 3) and study staff (*n* = 7), during which the CLDs were refined and strategies were identified. Concurrently, 19 participants completed an online survey assessing the perceived impact and feasibility of proposed strategies. A final plenary GMB session with PIs and study staff (*n* = 5) was conducted to gather feedback on the revised CLDs and prioritize strategies.

**Table 1:** Overview of study activities, participants, and outputs.

Activity	Participants	Output(s)
Analysis of semi-structured interview data	*n* = 48 principal investigators (PIs) and study staff representing 28 community-engaged research studies	Initial CLD
GMB session 1 (PIs)	*n* = 3 PIs with current community-engaged research experience	Refined CLDs across funding, institutional barriers, authentic community-academic partnerships Recommended strategies
GMB session 2 (study staff)	*n* = 7 study staff with current community-engaged research experience	
Perceived impact and feasibility survey	*n* = 19 participants from prior interviews and/or GMB sessions	Numeric rating of strategies on perceived impact and feasibility
Plenary session	*n* = 5 participants from prior interviews and/or GMB sessions	Feedback on CLDs Priority ranking of strategies

### 3.1 Initial CLD

The initial CLD shown in Figure 1A was drafted from semi-structured interview notes describing best practices and challenges in conducting inclusive CEnR. Three reinforcing feedback loops were identified. Reinforcing loop R1 illustrates the reciprocal relationship between the community involvement and researcher responsiveness, whereby increased community involvement leads to greater prioritization of community needs in research, which strengthens authentic researcher-community partnerships and further encourages engagement. Reinforcing loop R2 shows that authentic partnerships enable more community-based research and more meaningful outputs to the community, fostering continued engagement. Reinforcing loop R3 demonstrates how research activity can support the growth and long-term sustainability of community-based programs, contributing to improved community health outcomes and reinforcing community commitment to research participation.

In contrast, two balancing loops (B1 and B2) represent constraints within the system. These loops highlight how authentic partnerships require prioritization of community timelines, which often diverge from traditional grant cycles. This misalignment can reduce research productivity and funding availability, thereby limiting the scope and sustainability of CEnR.

The three boxed variables in Figure 1A – funding, institutional barriers, and authentic partnerships – were selected as focal domains for subsequent GMB activities, leading to additional feedback loops identified within each domain.

### 3.2 Funding feedback loops

Within the funding domain, two distinct sets of reinforcing loops were identified (Figure 1B). The first reinforcing loop R4 (orange) reflects the traditional research funding incentive structure. This loop reinforces research productivity through conventional outputs such as peer-reviewed publications and quantitative findings while de-emphasizing outputs that may be more relevant to communities, such as community forums, brief reports, or storytelling for advocacy. This dynamic perpetuates undervaluation of community-centered outcomes in academic and funding contexts.

A second set of reinforcing loops, shown in yellow (R5 and R6), act counter to R4, and represents how shifts in academic culture may be driven by greater dissemination and recognition of CEnR. As broader acceptance of community-based methods grows, non-traditional outputs like relationship-building and qualitative metrics may gain legitimacy across academic and funding institutions. In this way, academic culture shapes what research outputs are valued and rewarded. Although R4 likely exerts stronger influence in the current academic research climate, R5 and R6 represent a promising avenue for systemic transformation through realigned incentive structures.

### 3.3 Institutional barriers feedback loops

Within the institutional barriers domain, four additional sets of feedback loops were identified (Figure 1C). Reinforcing loop R7 (purple) illustrates how recruitment and retention of faculty engaged in community-based work help sustain research productivity and increase grant acquisition for CEnR projects. This dynamic is counterbalanced by two balancing loops (B3 and B4, black), which reflect the emotional toll of community-engaged scholarship. In high-pressure academic environments, burnout can lead to attrition and reduced productivity, weakening long-term partnerships, and institutional momentum for CEnR.

The reinforcing loops R8, R9, and R10 (red) highlight how growth in institutional knowledge of CEnR may catalyze shifts in academic culture. These shifts can lead to revised policies and processes that better support CEnR scholarship beyond traditional notions of rigorous clinical research. Reinforcing loop R11 (pink) further emphasizes the role of tenure and promotion in legitimizing community-based research. When CEnR work is recognized, it increases institutional support and funding opportunities and reinforces the value of community-engaged approaches.

### 3.4 Authentic partnerships feedback loops

Within the authentic partnership domain, three sets of feedback loops were identified (Figure 1D). Reinforcing loops R12 and R13 (light blue) show how sustained partnerships are supported by active community participation, responsiveness to community needs, inclusive representation, and mutual respect and trust. These elements underlie long-term collaboration and strengthen the legitimacy and relevance of CEnR outcomes. These loops also suggest that academic culture influences whether relationship-building, trust, and responsiveness to community priorities are recognized as important components of research.

Reinforcing loops R14 and R15 (dark blue) emphasize that research productivity and grant funding are also critical to sustain long-term community involvement by supporting researchers and ensuring fair compensation for community partners. Balancing loops B5 and B6 (black) highlight another important challenge: misaligned timelines between academic and community interest-holders can strain productivity and deplete financial resources, threatening long-term sustainability of CEnR programs.

### 3.5 Prioritized strategies

During the GMB sessions, participants identified 27 unique strategies to promote and sustain CEnR infrastructure, including five related to authentic partnerships, 11 related to funding, and 11 related to institutional barriers to CEnR (Table 2). Table 2 also presents priority rankings, mean feasibility and impact scores, and example feedback loops addressed by each strategy.

**Table 2:** Prioritized strategies by domain, mean feasibility and impact scores, and example feedback loops.

Strategy number	Level of influence	Strategy	Priority rank^a^	Mean feasibility score^b^	Mean impact score^b^	Example feedback loops
Domain: Authentic partnerships (*n* = 19 respondents)						
1	Researcher	Incorporate relationship-building into study staff roles and responsibilities and hire team members from the community	1st	4.33	4.75	R1, R12, R13
2	Funder and Institution	Provide resources and funding for multisector partnerships beyond the research study	2nd	2.73	4.64	R1, R13, R15
3	Researcher	Understand community culture and needs to inform research practices and priorities and to strengthen partnerships	3rd	4.42	4.67	R1, R12, R13
4	Researcher	Form Community Advisory Boards (CABs) and implement community-based participatory research (CBPR) best practices, including equitable compensation for community partners and collaboration beyond the research study	4th	4.25	4.67	R1, B5
5	Funder and Institution	Create an open environment for bidirectional dialog with community partners	5th	4.25	4.75	R1, R13
Domain: Funding (*n* = 15 respondents)						
6	Funder and Institution	Streamline and expand grant opportunities for community partners by simplifying application and reporting processes and allowing community partners to serve as primary applicants and recipients	1st	3.44	4.40	R4, R5, R6
7	Funder and Institution	Implement flexible research timelines and budgets to accommodate community-specific expenses (e.g., food, participant incentives) and the nonlinear nature of community engagement	2nd	3.35	4.67	R4, R5, R6
8	Institution	Streamline administrative accessibility for community-engaged research by simplifying subcontracting processes or reducing or removing indirect costs	3rd	3.25	4.60	R7, R8, R9, B3, B4
9	Funder	Diversify grant review panels to include reviewers with community-engaged research expertise	4th	4.11	4.67	R4, R5, R6
10	Funder	Broaden assessment criteria to evaluate community impact beyond quantitative metrics	5th	4.41	4.40	R4, R5, R6
11	Funder and Institution	Formalize community partner roles as multiple principal investigators and co-investigators	6th	3.53	4.27	R1, R2, R3
12	Institution	Extend pilot project duration to a minimum of 2 years	7th	3.68	4.07	R4, R5, R6
13	Funder	Strengthen community engagement requirements in funding opportunity announcements	8th	4.32	4.53	R4, R5, R6
14	Funder and Institution	Prioritize diverse funding sources (e.g., federal, private, non-profit)	9th	3.74	3.67	R4, R5, R6
15	Funder and Institution	Establish mentorship programs to foster relationships between researchers and community to conduct community-engaged research	10th	4.67	4.67	R1, R2, R3
16	Funder	Increase post-award flexibility in updates to project aims to reflect community priorities	11th	3.82	4.40	R4, R5, R6
Domain: Institutional Barriers (*n* = 13 respondents)						
17	Academic culture	Implement flexible research payment processes for community partners, such as simplifying reimbursement and billing processes	1st	3.58	4.69	R8, R9, B3, B4
18	Academic culture	Invest in community-engaged research infrastructure, including recognition of community engagement in promotion decisions and hiring dedicated personnel to support engagement activities	2nd	3.58	4.62	R8, R9, R10
19	Academic culture	Streamline administrative processes for community-engaged research by developing tailored Human Subjects Research (HSR) and ethics training for community partners and streamline IRB, legal, and finance systems to reduce burden on grantees	3rd	3.46	4.69	R8, R9, B3, B4
20	Academic culture	Develop specialized IRB review processes and guidelines that recognize the unique context and risk–benefit profile of community-engaged research	4th	3.38	4.54	R10, R11
21	Academic culture	Implement fair and inclusive hiring practices	5th	4.42	4	R7, R11
22	Researcher	Document and disseminate case studies describing the costs of community-engaged research	6th	4.62	4	R8, R9, R10
23	Academic culture	Build a network of community-engaged researchers	7th	4.54	4.31	R9, R10
24	Researcher	Conduct workshops and seminars showcasing successful community-engaged research studies	8th	4.77	4.15	R9, R10, R11
25	Academic culture	Foster positive relationships between Institutional Review Board (IRB) staff and researchers	9th	3.77	3.92	R8, R9, B3, B4
26	Academic culture	Ensure institutional leadership reflects communities served	10th	3.31	4	R1, R2, R12, R13
27	Researcher	Increase participatory and community-engaged research responsibilities, particularly in supervisory and leadership positions	11th	3.85	4.23	R10

^a^Priority rank was assigned within each domain.

^b^Scores ranged from 1 (not feasible or effective at all) to 5 (very feasible or effective), with higher scores indicating greater perceived feasibility or impact.

In the plenary session, the top-ranked strategies by domain were strategy 1 for authentic partnerships, incorporating relationship-building into study staff roles and responsibilities and hiring team members from the community; strategy 6 for funding, streamlining and expanding grant opportunities for community partners; and strategy 17 for institutional barriers, implementing flexible payment processes for community partners.

Figure 2 shows a scatterplot of mean perceived feasibility and impact scores for all strategies. Participants generally rated strategies related to authentic partnerships and funding as more feasible and impactful than strategies addressing institutional barriers. Eight strategies for authentic partnerships and funding were clustered in the upper-right portion of the figure, indicating relatively higher feasibility and impact compared to the other strategies. These included relationship-building and community hiring (strategy 1), understanding community context and needs to inform research (strategy 3), and having community advisory boards and following CBPR principles (strategy 4) by researchers; bidirectional communication (strategy 5) and mentorship to support CEnR (strategy 15) by funders and institutions; and diversification of grant review panels (strategy 9), broad evaluation criteria (strategy 10), and stronger CEnR requirements in grant applications (strategy 13) by funders.

**Figure 2:**
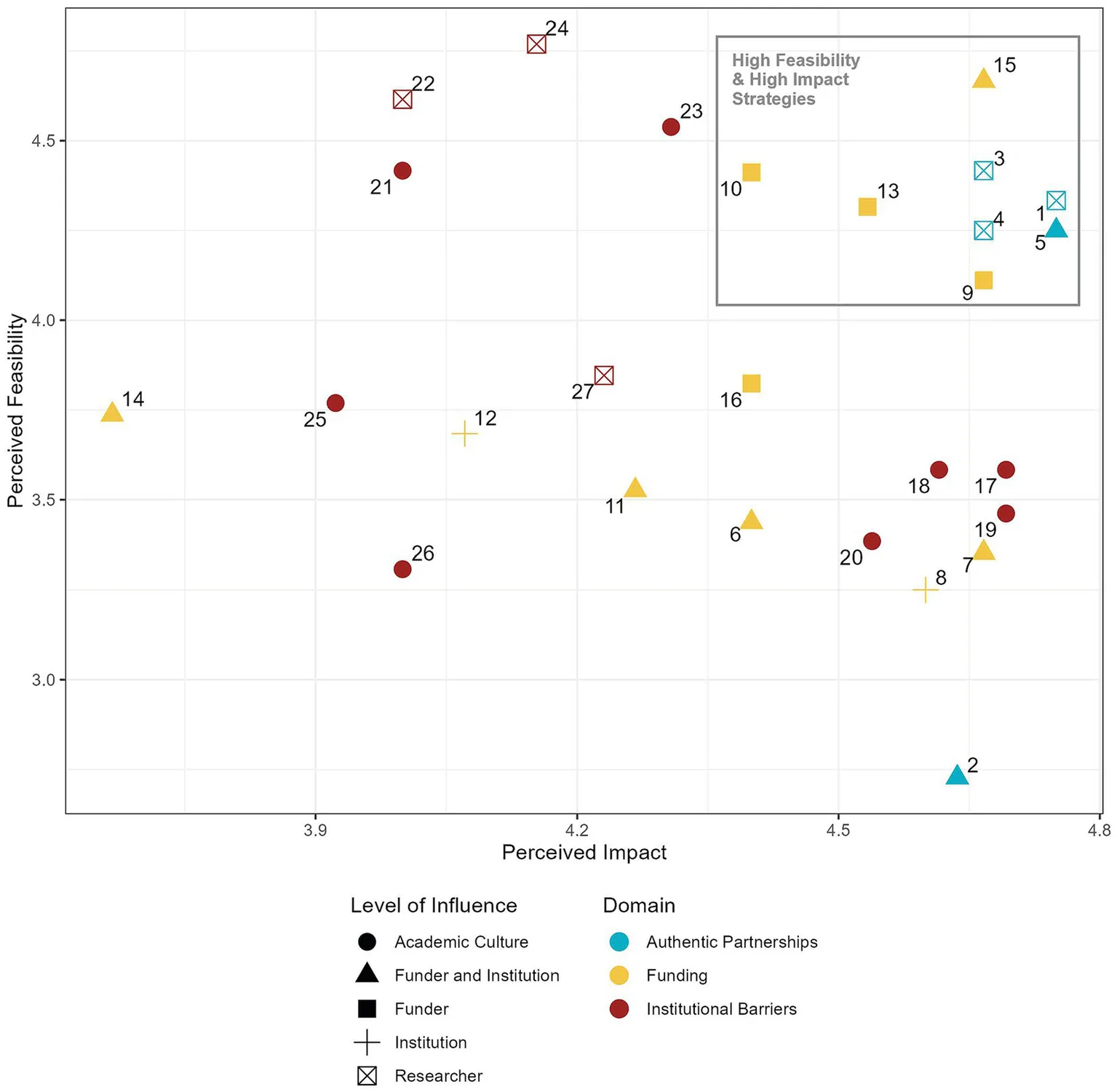
Perceived feasibility versus impact of strategies to strengthen community-engaged research infrastructure, by level of influence and domain. Note: Strategy numbers correspond to the prioritized strategies listed in Table 2. Strategies in the upper-right quadrant were interpreted as having relatively higher perceived feasibility and impact compared with other strategies. For ease of interpretation: **1**, incorporate relationship-building into study staff roles and responsibilities and hire team members from the community; **3**, understand community culture and needs to inform research practices and priorities and to strengthen partnerships; **4**, form Community Advisory Boards and implement community-based participatory research best practices; **5**, create environments that support bidirectional dialog with community partners; **9**, diversify grant review panels to include reviewers with community-engaged research expertise; **10**, broaden assessment criteria to evaluate community impact beyond quantitative metrics; **13**, strengthen community engagement requirements in funding opportunity announcements; and **15**, establish mentorship programs to foster relationships between researchers and community partners to conduct community-engaged research.

## 4 Discussion

This study developed comprehensive CLDs related to sustainability of academic CEnR and identified 27 strategies to shift system dynamics across researchers, funders, and institutions. Consistent with prior research, our interviews and initial CLD identified three central domains – funding, institutional barriers, and authentic community-academic partnerships (4, 19, 20). GMB workshops refined and validated the CLD and identified specific feedback loops and strategies that could strengthen academic systems supporting CEnR. We also assessed the perceived feasibility and impact of these strategies to identify short- and long-term opportunities for action.

This study extends the CEnR literature by moving beyond documentation of barriers to participatory development of actionable strategies (21). These findings also align with the literature on methodological and normative co-production in sustainability and environmental sciences, which has similarly argued for systemic changes within academia – including flexible and transparent funding mechanisms, revised tenure and promotion criteria, streamlined administrative processes, and stronger data governance and ethics review – to support equity-focused partnerships and justice-oriented knowledge production (10, 11).

Multi-pronged engagement of subject matter experts across interviews, GMB workshops, surveys, and a plenary session strengthened the relevance and applicability of the findings. The CLDs facilitated identification of leverage points for change and highlighted unintended consequences that linear frameworks may not capture, underscoring the need for coordinated efforts across researchers, institutions, funders, and academic culture. This study also adapted GMB and participatory system dynamics approaches to better generate insights that were immediately actionable rather than conceptual by using prior interviews to inform an initial CLD that was refined through workshops (16).

Similar to prior research, the feedback loops underscored how academic policies, incentive structures, and administrative processes both reinforce and constrain sustained CEnR (5, 19, 20). Reinforcing loops (R1-R3) emphasized how authentic partnerships, meaningful outputs, and community health outcomes can mutually strengthen one another, consistent with literature showing that CEnR depends on trust, bidirectional communication (22). At the same time, balancing loops (B1-B2, B5-B6) highlighted how administrative burdens, misaligned incentives and funding timelines diminish the capacity to sustain CEnR (4, 23). A systems framing explains the durability of engagement efforts – why efforts become difficult to maintain even when led by highly motivated individuals and why systems change is needed (24, 25). These findings suggest that CEnR is shaped by a broader academic research ecosystem where funders, academic institutions, community organizations, and policy conditions interact to influence partnerships, research feasibility, and what outputs are recognized. These findings suggest that sustaining CEnR requires structural change and a stronger commitment to community engagement at decision-making levels across the research ecosystem.

The CLD also identified multiple institutional leverage points to strengthen CEnR. Prior studies have shown how academic systems prioritize traditional research outputs over community engagement, creating downstream challenges for scholars who collaboratively work with communities (26, 27). Our findings similarly suggest that traditional productivity metrics (e.g., publications, quantitative outcomes) discourage relationship-building and community-preferred metrics (e.g., qualitative outcomes) that are essential to CEnR (R4), because of the mismatch between what outputs are valued by community versus what academia rewards (28). While academic cultural change is possible (R5-R6), institutional action is likely needed to change promotion and tenure policies, such as pilot funding for CEnR and promotion criteria that recognize community engagement and dissemination (29, 30). Without these changes, the misalignment between academia and community priorities is likely to persist.

Our findings also highlighted the emotional toll of CEnR in high-pressure academic environments. Burnout reduces faculty retention and productivity, weakening institutional capacity for CEnR (B3-B4). While emotional labor and burnout have often been discussed at the individual level (31, 32), our CLDs situated them within broader institutional dynamics. The reinforcing loops related to institutional recognition for CEnR suggest that revising administrative policies and promotion criteria could have downstream benefits for funding, productivity, and partnership sustainability. Put another way, having better systems in place can help to retain and encourage CEnR-focused researchers and importantly prevent burnout.

The prioritized strategies aligned closely with the feedback loops across the three domains. High-priority strategies (high-impact and high-feasibility) emphasized relationship-building, hiring from the community, responsiveness to community needs, greater funding flexibility, streamlining administrative processes, broader funding eligibility and acceptance fo diverse evaluation metrics as appropriate outcomes, and changes to administrative processes (e.g., payment methods, institutional review board review or CEnR human subjects studies) (33, 34). These findings point to the need for coordinated multi-level action to strengthen CEnR infrastructure through shared decision-making, more equitable research processes and research ownership, and greater recognition and higher valuation of community-engaged scholarship (35, 36).

These findings are particularly relevant in the current funding context, as major health research funders are increasingly emphasizing patient, community, and interest-holder engagement in research. However, our findings suggest that despite this growing methodological priority, existing academic funding and institutional infrastructures remain poorly aligned with what is required to support and sustain CEnR. The prioritized strategies identified in this study offer practical opportunities to better align these systems with the stated funder priorities, while also recognizing that workshop participation included a subset of interview participants and therefore may not represent the full range of perspectives and strategies on CEnR system change. These strategies also suggest that an adapted participatory GMB approach may provide a feasible model for examining complex health research systems and generating model-informed strategies that are actionable in real-world implementation and practice (16).

### 4.1 Strengths and limitations

The participatory GMB process was a tool for shared understanding and co-development of strategies to improve CEnR sustainability in the academic context. A key strength of this study was its multi-pronged design, which captured diverse expert perspectives across roles (i.e., PI, research staff) and focus populations. This approach enabled participants to identify shared and distinct challenges and to co-develop and prioritize strategies. The systems framing also strengthens the study by moving beyond documentation of barriers to identify practical leverage points to support CEnR sustainability.

Study limitations include the limited generalizability of findings due to relatively small number of participants in the GMB workshops and plenary session, particularly relative to the original interview sample, which may have limited the breadth of perspectives and completeness of feedback and the identified strategies. The study focused on three domains –funding, institutional barriers, authentic partnership–which may have overlooked other factors influencing CEnR sustainability, and the CLD represents a cross-sectional model of current CEnR systems. However, the multi-stage study activities, consistency of themes across the interviews and workshops, and inclusion of participants with varied roles, topical expertise, geographic locations, and CEnR experience, providing diverse perspectives to inform recommendations across contexts.

We also recognize the limitation of not inviting research community partners to share their perspectives on CEnR. However, because the nexus of influence can be most strongly shaped starting with the academic setting, we opted to focus on this area as a maximal catalyst for change. More importantly, we want to advocate for shifts in academic culture where the onus is placed on researchers and research institutions to better accommodate community partners and participant preferences versus the framing of participant- and community partner-level barriers and challenges that keep them from participating and partnering in research (37). Future research could refine these CLDs by incorporating community perspectives and the perspectives of institutional leaders to develop additional recommendations and better understand why CEnR reforms often fail to gain traction in academic settings.

## 5 Conclusion

This study identified key drivers and leverage points for CEnR in academic settings. Barriers to CEnR arise from interacting dynamic factors across academic culture, funding structures, and community-academic partnerships. Prioritized strategies highlight opportunities for researchers, institutions, and funders to strengthen the durability and long-term impact of CEnR.

## Data Availability

The datasets presented in this article are not readily available because they were generated through group model building sessions and ranking exercises and may contain information that could compromise participant confidentiality. Requests to access the datasets should be directed to LĐ, Lan.Doan@nyulangone.org.

## Footnotes

1 https://vensim.com/

2
https://www.r-project.org/
