# Supplementary material for: Identifying leverage points to sustain community-engaged research infrastructure in academic systems: a participatory systems science approach
Source: Front Public Health. 2026 Oct 21;14:1970793. doi: 10.3389/fpubh.2026.1970793 (PMC13731354; doi:10.3389/fpubh.2026.1970793)
Supplement: Supplementary file 1 [file Data_Sheet_1.docx]

Supplementary Material

# 1 Supplementary Data

## 1.1 Supplementary Data 1. Guide to reading causal loop diagrams provided to participants

**How to Read a Causal Loop Diagram**

**Causal loop diagrams (CLDs)** are a visual representation of hypothesized causal relationships between variables and feedback structures within a system.

CLDs are made up of a core set of building blocks: **variables**, **arrow linkages**, and **feedback loops**, which can be connected to form more complex system structures. **Arrow linkages** between variables represent hypothesized causal relationships. A **feedback loop** is represented by a closed series of arrow linkages which can produce either **reinforcing** or **balancing** effects on system behavior.

A. A **positive (+) linkage** indicates a change in the first variable produces a change in the same direction of the variable that follows. *For example, increased access to healthy foods leads to increased diet quality, and conversely, decreased access to healthy foods leads to decreased diet quality.*
B. A **negative (−) linkage** indicates a change in the first variable produces a change in the opposite direction of the variable that follows. *For example, increased food costs lead to decreased access to healthy foods, and decreased food costs lead to increased access to healthy foods.*
C. For some variables, the effect on the variable that follows happens with some **time delay**, as depicted by hash marks intersecting an arrow linkage.
D. **A reinforcing feedback loop** contains an even number of (or zero) negative linkages and tends to accelerate the effects of disruptions to the system by leading to exponential growth or exponential decline of variables. Reinforcing loops can produce favorable outcomes (*e.g., an increase in access to healthy food increases consumption of and demand for healthy foods, which increases supply and access*) or unfavorable outcomes (*e.g., a decrease in access to healthy food decreases consumption of and demand for healthy foods, further lowering supply and access*).
E. A **balancing feedback loop** contains an odd number of negative linkages and tends to stabilize the effects of disruptions to the system, bringing variables into steady states. *For example, an increase in government funds for nutrition programs that increase access to healthy foods may lead to healthier eating and a decline in diet-related disease. When diet-related disease rates are low, funding may also decline due to lack of perceived need for nutrition programs.*

Adapted from: Chebli, P., Đoàn, L.N., Thompson, R.L. *et al.* Identifying opportunities for collective action around community nutrition programming through participatory systems science. *Cancer Causes Control* 34, 1043–1058 (2023). <https://doi.org/10.1007/s10552-023-01751-6>
